# Facile Preparation and Characterization of Polyaniline and CeO_2_ Co-Decorated TiO_2_ Nanotube Array and its Highly Efficient Photoelectrocatalytic Activity

**DOI:** 10.1186/s11671-019-2897-y

**Published:** 2019-02-19

**Authors:** Chunmao Chen, Danchen Zhao, Qingxiang Zhou, Yalin Wu, Xianqi Zhou, Hongyuan Wang

**Affiliations:** State Key Laboratory of Heavy Oil Processing, State Key Laboratory of Petroleum Pollution Control, China University of Petroleum Beijing, Beijing, 102249 China

**Keywords:** TiO_2_ nanotube arrays, Polyaniline, Cerium dioxide, Tetrabromobisphenol A, Photoelectrocatalytic activity

## Abstract

In the present work, polyaniline and CeO_2_ co-decorated TiO_2_ nanotube arrays (PANI/CeO_2_/TiO_2_ NTAs) were facilely prepared by an electrochemical method. The as-prepared materials were characterized by scanning electron microscopy (SEM), an X-ray diffractometer (XRD), and energy-dispersive X-ray spectroscopy (EDS). The photoelectrocatalytic activity of as-prepared materials was investigated with tetrabromobisphenol A (TBBPA) as the target analyte, and the data showed that PANI/CeO_2_/TiO_2_ NTAs resulted in much higher photoelectrocatalytic efficiency than that of other materials. Under optimal conditions, the degradation rate of TBBPA reached a maximum value over 96% in 120 min under simulated solar irradiation. The results indicated that CeO_2_ and PANI co-modified TiO_2_ NTAs could narrow the band gap, expand the response from ultraviolet (UV) to visible region, increase the amount of active free radicals, inhibit the recombination rate of electron-hole pairs, and finally enhance the degradation efficiency towards TBBPA owing to the presence of Ce^3+^/Ce^4+^ and PANI. Moreover, the degradation reaction followed the first-order kinetics, and degradation rates of the repeated experiments were all over 92% for ten runs. All these results indicated that this novel catalyst earned great potential as a powerful photoelectrocatalyst for the removal of TBBPA and other pollutants.

## Introduction

Rapid development of industrialization all over the world led to the generation of various pollutants, which contain different kinds of toxicants including inorganic or organic pollutants. The toxic effect of these pollutants has thrown a serious threat on environment and human health, and absorbs much more attention. Therefore, more attention has been put on development of efficient and clean degradation technologies for these contaminants. Photocatalysis, a convenient, economical, and enhanced conventional treatment technology, has been an important technology to remove these organic pollutants [1]. The core part is the photocatalyst when this technology is involved. Recently, heterogeneous photocatalysts, especially TiO_2_ and related materials, have received most attention due to their low-cost, stable chemical, non-toxic, and narrow-band-gap properties. TiO_2_-based catalytic materials have been proved to be used to effectively remove the toxic and hazardous organic pollutants in contaminated air and water, which is of great significance for the environmental protection [2–4]. Tetrabromobisphenol A (TBBPA) is one of brominated flame retardants (BFRs) and accounts for approximately 60% of the total BFR market, which are commonly used in clothes, toys, electronics, plastics, motor vehicles, and textiles to reduce flammability. TBBPA is found in various matrices such as water, soil, air, and sediment, and even human blood and breast milk [5, 6]. It is reported that TBBPA affects humans’ health seriously as an endocrine disruptor [7]. Therefore, to develop rapid degradation technologies of TBBPA is necessary for both environmental monitoring and human health protection.

Now, many studies have revealed that TiO_2_ has its own weakness. Its relatively wide band gap (~ 3.20 eV) is the main limitation for its industrial application, which means that TiO_2_ can only be activated by irradiation with a wavelength less than 387 nm and is sensitive to UV light [8–11]. A lot of research efforts, such us sensitization, doping rare metal ion, doping metalloid, and coupling semiconductor [12–16], have been made all around the world in order to extend the application of TiO_2_. It has been proved that noble metals of Au, Ag, Pt, and Pd deposited onto the surface of TiO_2_ can modify the surface properties of the material and enhance the catalytic capability [17, 18]. On the other hand, metal oxide may be another effective functionalized modification material. The band gap of CeO_2_ is approximately 2.92 eV, and the variable valences of Ce such as Ce^3+^ and Ce^4+^ make CeO_2_ possess the excellent ability in transferring electrons and hindering the recombination of photogenerated electron-hole pairs, which make CeO_2_ become an attractive modification material to enhance the photocatalytic capability of TiO_2_ [19–21]. In addition, CeO_2_ doped in the TiO_2_ NTAs can produce a certain amount of hydroperoxy radical (HO_2_•), which is one of the main active species in the degradation procedure. In spite of these advantages, CeO_2_/TiO_2_ catalysts hardly show much higher photocatalytic activity due to its low specific surface area and mass-transfer limitation of target pollutants. Polyaniline (PANI) has exhibited its good merits and achieved many applications. Currently, some researchers synthesized PANI/TiO_2_ nano-materials and confirmed the outstanding stability of them due to facile synthesis, low-cost, chemical stability, and charge storage capacity [22, 23]. Moreover, PANI could be able to absorb more visible light photons and inject electrons to the conduction band (CB) of TiO_2_, which would promote the photocatalytic process [23].

However, to the best of our knowledge, less attention has been paid on a TiO_2_ material doped by CeO_2_ and coated by PANI simultaneously for photodegradation of TBBPA. In this work, PANI/CeO_2_/TiO_2_ NTAs were designed to graft the superiority of CeO_2_ and PANI on the defective TiO_2_ NTAs. As expected, PANI/CeO_2_/TiO_2_ NTAs exhibited highly improved photoelectrodegradation activity as compared to pure TiO_2_ NTAs, CeO_2_/TiO_2_ NTAs, and PANI/TiO_2_ NTAs. Microstructure and morphology of the PANI/CeO_2_/TiO_2_ NTAs were characterized by scanning electron microscopy (SEM) and energy-dispersive X-ray spectroscopy (EDS). Some factors that influenced the degradation efficiency including the loading amount of CeO_2_/PANI, annealing temperature, pH value, and hole scavengers were investigated. A preliminary mechanism study indicated that active oxyradicals such as HO_2_• and HO•, which formed via synergetic effect of PANI, CeO_2_, and TiO_2_, were of a great contribution to remove TBBPA.

## Materials and Methods

### Materials

All raw materials used were of analytical grade except methanol, and all solutions in the synthesis and treatment processes were prepared with deionized water. Titanium films (99.6% purity) were purchased by Northwest Institute for Non-ferrous Metal Research, China. Aniline was purchased from JinKe Fine Chemical Institute, China. Isopropyl alcohol was obtained from Tianjin Guangfu Technology Development Co. Ltd., China. NaF, H_3_PO_4_, HCl, and acetone were obtained from Beijing Chemical Works, China. Na_2_SO_4_, CeCl_3_·7H_2_O, and TBBPA were purchased from Aladdin Chemistry Co. Ltd., China. High-performance liquid chromatography (HPLC) methanol was purchased from Oceanpak Alexative Chemical, Sweden. All chemicals were used as received without further purification.

### Preparation of TiO_2_ NTAs

Ti foils were polished by different abrasive papers in order to remove impurity and obtain mirror surface. The TiO_2_ NTAs (20 × 25 × 0.2 mm) were fabricated by an anodization method. Then the Ti foils were cleaned by acetone, isopropyl alcohol, and methanol in an ultrasonic bath. The cleaned foils were anodized with the mixture of 0.5 M H_3_PO_4_ and 0.14 M NaF as the electrolyte in a two-electrode cell with Pt as the counter electrode at 20 V for 30 min. The obtained foils were rinsed with distilled water and dried under ambient conditions. After calcined at 500 °C for 2 h in muffle furnace, the NTAs were obtained. Eventually the NTAs were cleaned with deionized water and dried in air at room temperature.

### Preparation of CeO_2_/TiO_2_ NTAs and PANI/CeO_2_/TiO_2_ NTAs

The appropriate cerium content was deposited on the TiO_2_ films by a galvanostatic method. CeO_2_/TiO_2_ NTAs were prepared in a three-electrode system by an electrochemical way using uncalcined TiO_2_ NTAs as the working electrode, platinum foil as the counter electrode, and saturated calomel electrode (SCE) as a reference electrode. The plating solution is 0.025 M CeCl_3_ in deionized water [24]. The samples were immersed in the plating solution for 1 h before the deposition process. The electrodeposition current was set as 0.3 mA for 15 min, so that the amount of Ce deposited on the NTAs could be controlled. Then the deposited films were washed with deionized water and dried at room temperature. The as-prepared samples were annealed in a muffle furnace at different temperature for 2 h to convert Ce into CeO_2_ and form anatase crystal.

PANI/CeO_2_/TiO_2_ NTAs were synthesized by the galvanostatic method in a three-electrode system as well. The as-prepared CeO_2_/TiO_2_ NTA electrode was put into a solution of 0.5 M Na_2_SO_4_ and 0.2 M aniline, and a constant anodic current of 0.3 mA was loaded in a CHI660E electrochemical workstation. The polyaniline coating was adhered to the surface of the CeO_2_/TiO_2_ NTA substrate. The loading amount of PANI could be controlled by conduction time. After being cleaned and dried, PANI/CeO_2_/TiO_2_ NTAs were achieved.

### Characterization

The morphology of samples was characterized by a SU8000 scanning electron microscope (SEM) at an acceleration voltage of 5 kV. Chemical compositions were obtained by an energy-dispersive X-ray detector (EDAX, America) equipped with a scanning electron microscope. The crystal phases were examined by an X-ray diffractometer (XRD, Bruker D8 Advance, Germany).

### Photoelectrocatalytic Activity of CeO_2_/TiO_2_ and PANI/CeO_2_/TiO_2_ NTAs

The photoelectrocatalytic (PEC) activity of the two as-prepared NTAs was investigated with TBBPA as the model compound. The PEC degradation of 10 mg L^− 1^ TBBPA was performed in a regular quartz beaker using a three-electrode system with a 500-W xenon lamp with an optical filter as a simulative sunlight source. The luminous intensity of the Xe lamp was 120 mW/cm^2^. Moreover, 0.05 M Na_2_SO_4_ was added as the supporting electrolyte in the reaction beaker. Twenty microliters reaction solution was quickly taken out and analyzed on a LC-20AT high-performance liquid chromatograph (HPLC) every 15 min in the PEC degradation experiment process. The HPLC was composed of a LC-20AT pump, a separation column (Agilent SB-C18, 150 × 4.6 mm, 5 μm), and a VWD detector (SPD-20A). The mobile phase consisted of methanol and water (85: 15, *v*/*v*), and the flow rate was set at 1 ml min^− 1^.

## Results and Discussion

### Material Characterization

**Fig. 1:**
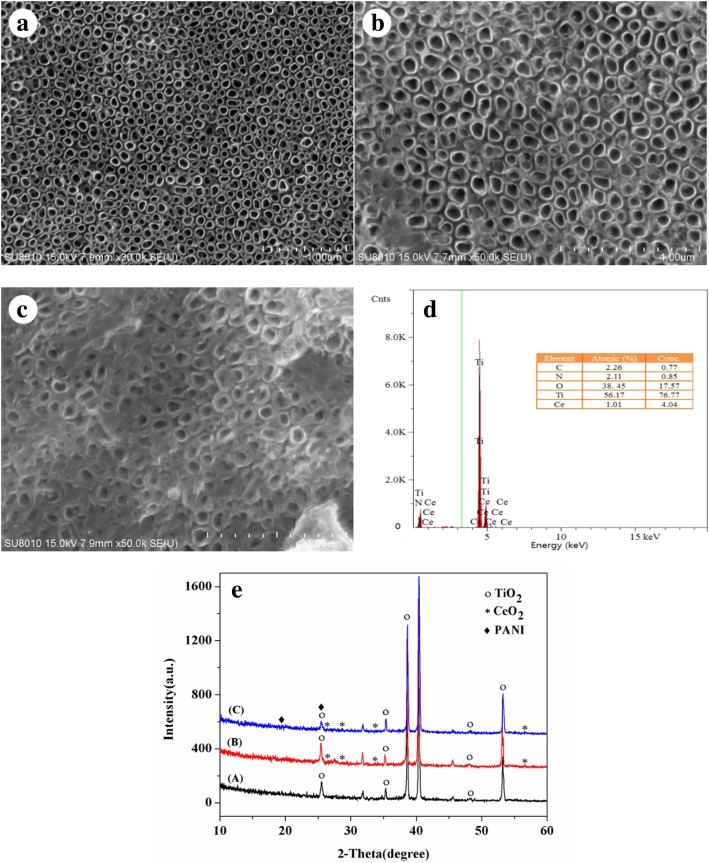
SEM images of TiO_2_ NTAs (**a**), CeO_2_/TiO_2_ NTAs (**b**), PANI/CeO_2_/TiO_2_ NTAs (**c**), and the EDS spectra of PANI/CeO_2_/TiO_2_ NTAs (**d**), and XRD patterns of TiO_2_ NTAs (A), CeO_2_/TiO_2_ NTAs (B), and PANI/CeO_2_/TiO_2_ NTAs (C) (**e**)

The surface morphologies of the prepared TiO_2_ NTAs, CeO_2_/TiO_2_ NTAs, and PANI/ CeO_2_/TiO_2_ NTAs were examined by SEM and shown in Fig. 1. The bare TiO_2_ NTAs have a clear microstructure and are composed of well-ordered, uniform, and high-density TiO_2_ nanotubes with pore sizes ranging from 90 to 110 nm and wall thickness of around 5 nm (Fig. 1a). After electro-deposition of CeO_2_ on the TiO_2_ NTAs, some CeO_2_ nanoparticles were uniformly formed on the top surface of TiO_2_ NTAs (Fig. 1b). It can be inferred that there should be part of CeO_2_ NPs in the tubes. Figure 1c shows that a porous and laminar PANI film was tightly adhered to the CeO_2_/TiO_2_ substrate after electro-deposition treatment with pore sizes ranging from 50 to 70 nm and wall thickness of about 40 nm. At the optimum anode current, aniline concentration, and deposition time, uniform PANI grew at the top of the tube walls [25]. The polymerization of aniline monomers occurred along the wall of the CeO_2_/TiO_2_ NTAs, proceeding into the pores until they were coated to the top surface of the NTAs. At the same time, the polymerization occurred among the proximate tube walls, leading to the growth of planar sheets of PANI. The existence of Ti, C, N, O, and Ce elements proved that PANI and CeO_2_ were modified on the TiO_2_ films (Fig. 1d). Further, the EDS results of the PANI/CeO_2_/TiO_2_ NTAs showed that the amount of N and Ce were about 2.11 at.% and 1.01 at.%, respectively. Figure 1e shows the X-ray diffraction pattern of TiO2 NTAs, CeO_2_/TiO_2_ NTAs, and PANI/CeO_2_/TiO_2_ NTAs. The peaks at the 2*θ* of 25.5°, 38°, 48°, and 53.3° were the peaks of the (110), (103), and (105) diffractions of anatase-phase TiO_2_, respectively. The peaks at 40.5° and 56.6° would be assigned to the titanium substrate. The little peaks of 2*θ* at 28.6° and 33.0° indicate the crystal phase of CeO_2_. But no significant difference was found between CeO_2_/TiO_2_ NAs and PANI/CeO_2_/TiO_2_ NAs, which may be due to the fact that only a quite low amount of PANI was loaded and which results in poor response in the XRD patterns.

### Comparison of Different Catalysts of Photoelectrocatalytic Degradation of TBBPA

**Fig. 2:**
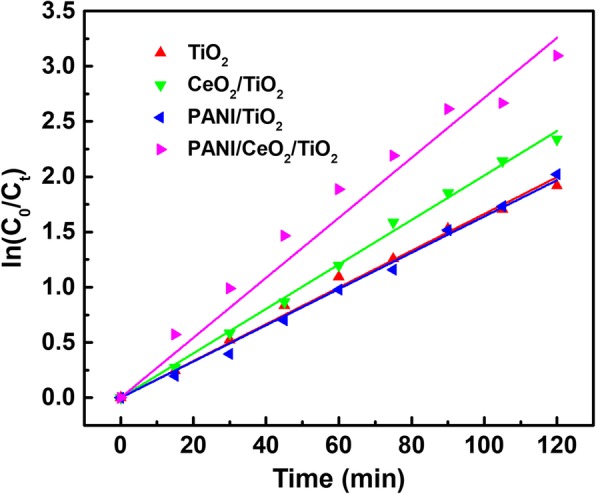
Photoelectrocatalytic degradation of TBBPA solution over the pure TiO_2_ NTAs, CeO_2_/TiO_2_ NTAs, PANI/TiO_2_ NTAs, and PANI/CeO_2_/TiO_2_ NTAs. The geometric surface area of TiO_2_ electrode was 2.0 × 2.5 cm^2^. Initial concentration of TBBPA: 10 mg L^− 1^, volume: 50 mL, electrolyte: 0.05 M Na_2_SO_4_, bias potential: 9 V

In order to assess the photoelectrocatalytic activity of the catalysts, the degradation rate of TBBPA with different catalysts was measured, and the reaction solution was 0.05 mol L^− 1^ Na_2_SO_4_ solution containing 10 mg L^− 1^ TBBPA and the external potential was 9.0 V. Figure 2 shows the degradation rates of TBBPA after 2 h with pure TiO_2_ NTAs, CeO_2_/TiO_2_ NTAs, PANI/TiO_2_ NTAs, and PANI/CeO_2_/TiO_2_ NTAs. The experimental results indicated that the photoelectrocatalytic efficiency of PANI/CeO_2_/TiO_2_ NTAs was the highest. The degradation efficiencies on TiO_2_ NTAs, CeO_2_/TiO_2_ NTAs, PANI/TiO_2_ NTAs, and PANI/CeO_2_/TiO_2_ NTAs were 85.34%, 90.33%, 86.78%, and 93.98%, respectively. Compared with TiO_2_ NTAs, the degradation efficiency of PANI/CeO_2_/TiO_2_ NTAs increased markedly by nearly 8.64%, and which also proved that the modification of CeO_2_ and PANI enhanced the photoelectrocatalytic capacity of the TiO_2_ NTAs. These results were approximately in agreement with the reported results [26].

### Influence of Preparation Parameters on the Photoelectrocatalytic Degradation of TBBPA

A series of synthesis and degradation experiments were performed to investigate the factors influencing the photocatalytic degradation of TBBPA and obtain the optimal synthetic parameters of PANI/CeO_2_/TiO_2_ NTAs in a three-electrode system including the CeO_2_ loading amount, PANI loading amount, and annealing temperature.

**Fig. 3:**
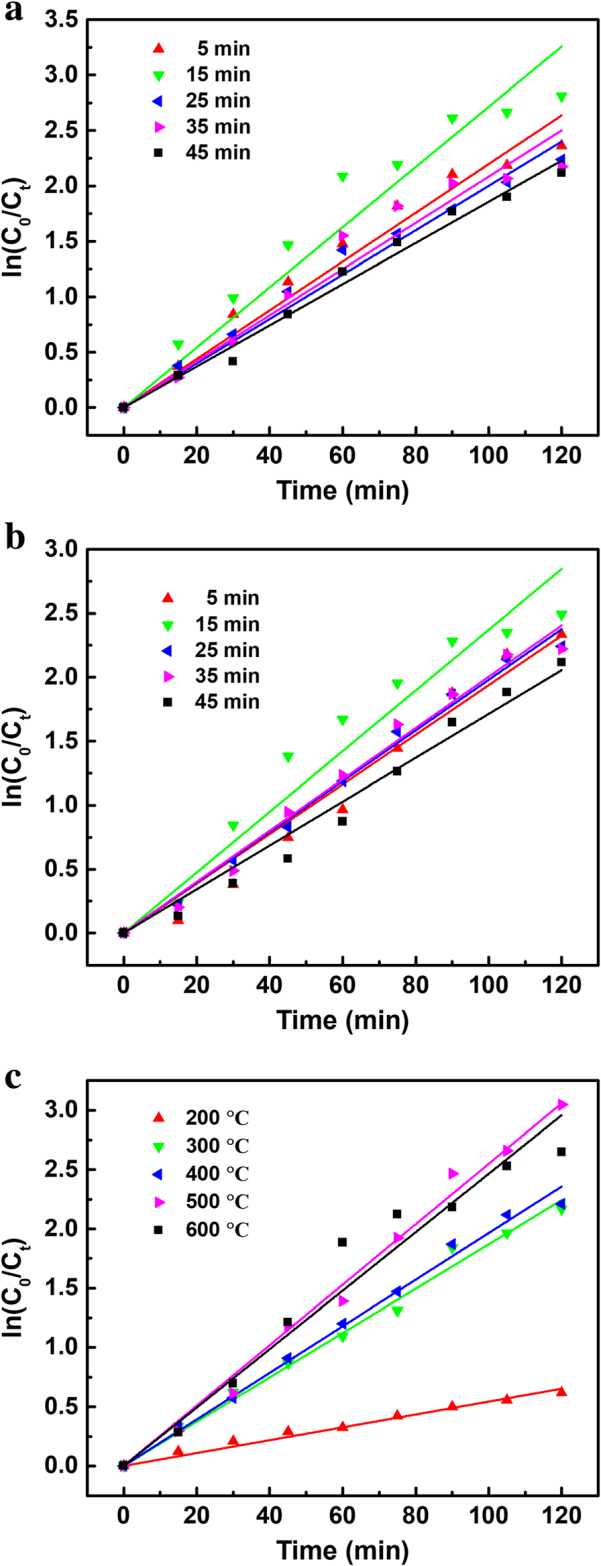
Effects of **a** CeO_2_ loading amount, **b** PANI loading amount, and **c** annealing temperature. The geometric surface area of TiO_2_ electrode was 2.0 × 2.5 cm^2^. Initial concentration of TBBPA: 10 mg L^− 1^, volume: 50 mL, electrolyte: 0.05 M Na_2_SO_4_, bias potential: 9 V

Figure 3a shows the effect of different CeO_2_ loading amounts on the photoelectrocatalytic performance of TiO_2_ film towards TBBPA degradation. Under the same conditions, the amount of CeO_2_ on the films was controlled by the deposition time. During the 120 min illumination, the CeO_2_/TiO_2_ NTAs with a deposition time of 15 min exhibited the highest photoelectrocatalytic activity, while the CeO_2_/TiO_2_ with a deposition time of 45 min exhibited the lowest photoelectrocatalytic activity. After the introduction of CeO_2_, the photoelectrocatalytic capabilities of all as-prepared CeO_2_/TiO_2_ NTAs were enhanced definitely compared with the bare TiO_2_ NTAs.

As reported, CeO_2_ could trap the photogenerated electrons and the Ce^4+^ was reduced to Ce^3+^ because of the coexistence of the Ce^4+^ and Ce^3+^ in the CeO_2_/TiO_2_ composite. Then the Ce^3+^ was prone to be oxidized back to Ce^4+^ by the adsorbed oxygen in the water. Meanwhile, the chemisorbed oxygen was reduced to superoxide radicals (O_2_^−^). Thus CeO_2_ altered the photogenerated electron-hole pair recombination rate in a certain extent, which gave rise to improved photoelectrocatalytic degradation efficiency of TBBPA [27]. It can be seen in Fig. 3a, the highest degradation rate of TBBPA was 93.98% when the deposition time of CeO_2_ reached 15 min. However, when the deposition time increased up to 45 min, the degradation rate of TBBPA was only 87.96%. This fact suggested that excessive amount of CeO_2_ coated on the surface of the composite will occupy some of the active sites of NTAs or act as a new recombination center of the electron-hole pairs to hinder the degradation of TBBPA.

PANI has been an important conductive polymer applied in the electro-optic field due to its good conductivity, charge storage capacity, and oxidation-reduction ability. Moreover, its electrochromic performance can enhance the absorption of the visible light and separation of electron-hole pairs rapidly, which can induce more photogenerated electrons [27, 28]. So, decorating TiO_2_ NTAs with PANI is a positive attempt to enhance the photoelectrocatalytic performance. A series of degradation experiments were performed to investigate the optimum loading amount of PANI in a three-electrode system, and the results were shown in Fig. 3b. The amount of PANI on the films was controlled by the electro-deposition time under the optimal immobilizing conditions. The results exhibited that the degradation rate of TBBPA firstly increased along with the increase of electro-deposition time, but decreased after 15 min. It was found that the CeO_2_/TiO_2_ NTAs coated with PANI for 15 min exhibited the highest photoelectrocatalytic degradation efficiency towards TBBPA. The curvilinear trend of degradation rate suggested that the existence of PANI could enhance the photoelectrocatalytic performance of TiO_2_ NTAs under simulated sunlight irradiation, and an excess amount of PANI coated on the NTAs would inhibit the irradiation absorbance of NTAs and influence the good contact with TBBPA of TiO_2_. Hence, electro-depositing TBBPA for 15 min, which could keep a highest light use ratio, was applied in the subsequent experiments.

Annealing temperature is one of the important factors in the synthesis of nanomaterials, which can easily change the crystalline phase of the materials and alter the photoelectrocatalytic activity by a large margin. Besides, after annealing, Ce ions are oxidized to CeO_2_, which will also make a positive contribution to the catalytic reaction. Figure 3c shows the photoelectrocatalytic performance of PANI/CeO_2_/TiO_2_ NTAs towards TBBPA at different annealing temperature. It can be seen that the degradation efficiency of TBBPA increased when the annealing temperature increased from 200 to 500 °C. It is known that anatase TiO_2_ exhibited a higher photocatalytic activity than that of other phases (amorphism and rutile). Figure 3c indicates that the TiO_2_ was mainly amorphous when annealing temperature was 200 °C, the amorphous TiO_2_ could gradually convert into anatase when annealing temperature was 500 °C, which accounted for the increase of the degradation efficiency of TBBPA. Rutile phase appeared and the degradation efficiency declined slightly when the annealing temperature reached 600 °C as reported [29].

### Optimization of Photoelectrocatalytic Degradation of TBBPA with PANI/CeO_2_/TiO_2_ NTAs

**Fig. 4:**
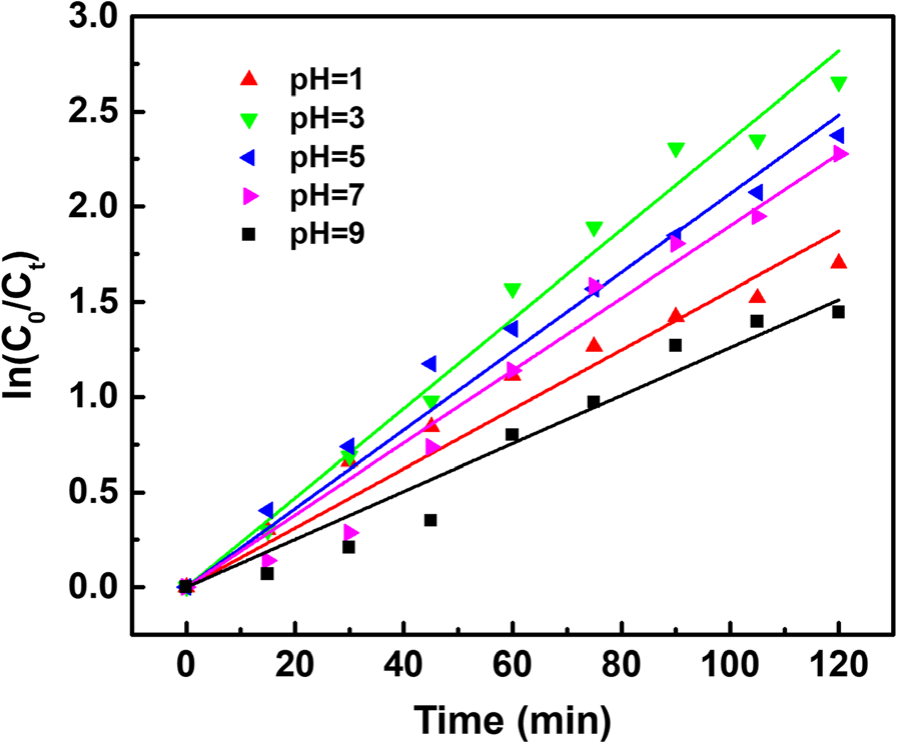
The effect of different pH on TBBPA degradation efficiency in photoelectrocatalytic process under simulated daylight irradiation. The geometric surface area of TiO_2_ electrode was 2.0 × 2.5 cm^2^. Initial concentration of TBBPA: 10 mg L^− 1^, volume: 50 mL, electrolyte: 0.05 M Na_2_SO_4_, bias potential: 9 V

The pH value will alter the ionization state of organic compound, the surface property of catalyst as well as the reaction matrices. It is believed that pH of the solution can influence the formation rate of hydroxyl radicals and other reactive oxygen species responsible for the pollutant degradation. The effect of initial pH value on the degradation efficiency is shown in Fig. 4. It was found that 92.96% TBBPA was photoelecetrodegraded after 120 min under simulated solar irradiation at pH of 3. Alkaline condition seemed to exhibit much stronger inhibition effect than that of acidic condition. The photogenerated electron-hole pairs were generated from the PANI/CeO_2_/TiO_2_ NTA sheet under simulated solar irradiation, which led to the reduction and oxidization of cerium and formation of •O_2_^−^. The •O_2_^−^ could not only react with H^+^ and then produce HO_2_• and •OH, two kinds of strong oxidative and reactive species, but also directly react with TBBPA. At the same time, it is reported that PANI has the higher catalytic activity in the acid solution. As a consequence, a low pH value is favorable for the formation of HO_2_• and •OH, while a high pH value could lead to an inhibition to the generation of HO_2_• and •OH, reducing the photoelectrocatalytic degradation efficiency.

**Fig. 5:**
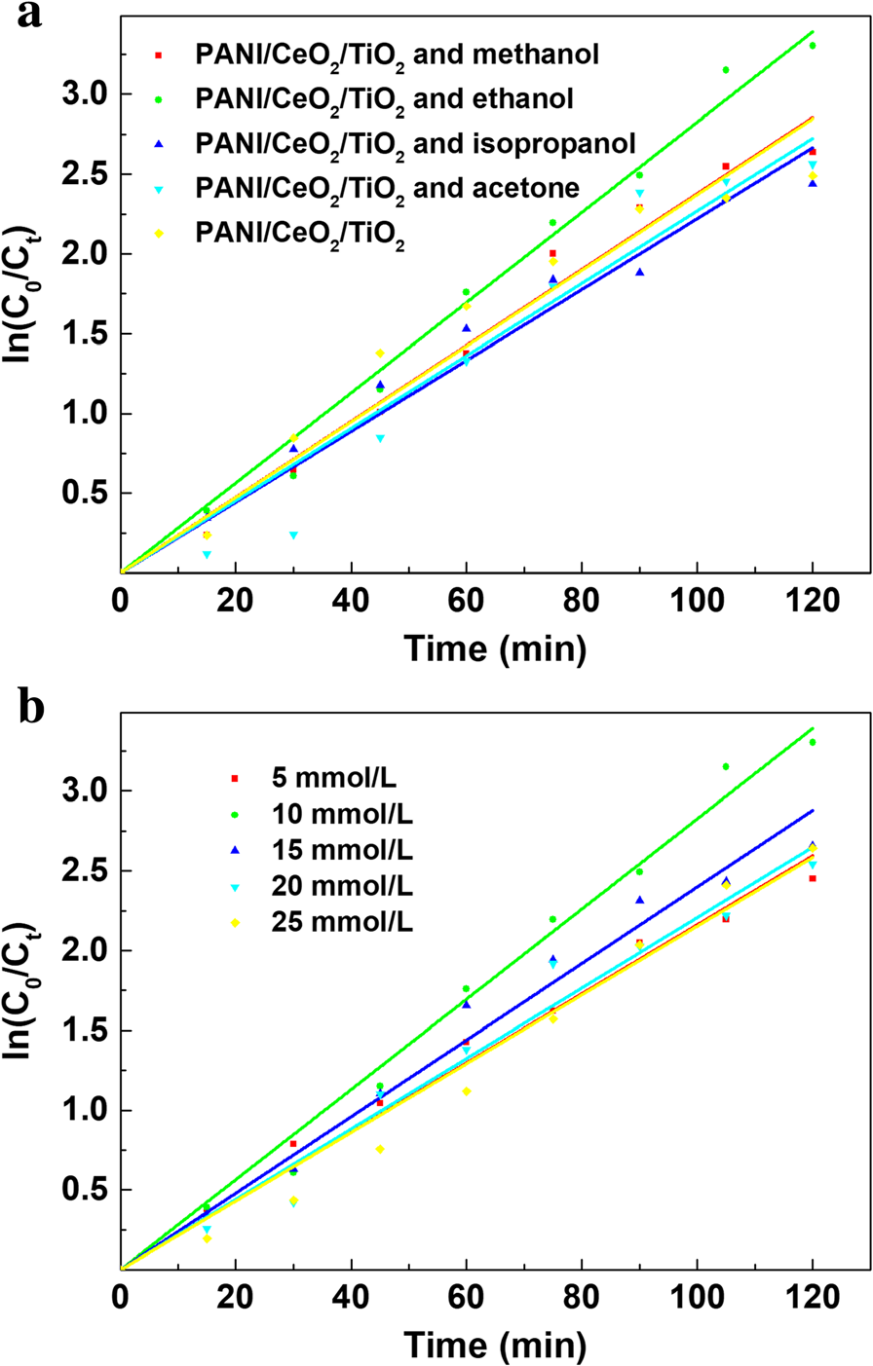
The effect of different hole scavengers (**a**) and ethanol amount (**b**) on the kinetics of TBBPA degradation. The geometric surface area of TiO_2_ electrode was 2.0 × 2.5 cm^2^. Initial concentration of TBBPA: 10 mg L^− 1^, volume: 50 mL, electrolyte: 0.05 M Na_2_SO_4_, bias potential: 9 V, pH: 3

In the photoelectrocatalytic degradation procedure, the recombination of electrons and electron-hole pairs significantly affected the catalytic performance of TBBPA. It has been reported that the hole scavenger could enhance the degradation ability of the TiO_2_ catalytic materials [30, 31]. In general, it is beneficial to add a hole scavenger to inhibit the recombination of electrons and electron-hole pairs and further achieve high photoelectrocatalytic activity. Compared with PANI/CeO_2_/TiO_2_ NTAs, four different hole scavengers (methanol, ethanol, isopropanol, and acetone) were investigated, and the results were presented in Fig. 5a. The presence of ethanol resulted in the highest degradation efficiency of TBBPA (96.32%), yet the degradation efficiencies of TBBPA using other hole scavengers (isopropanol and acetone) slightly reduced the efficiency compared to the blank controls. In addition, methanol had no influence on the degradation of TBBPA. Since the degradation rate constant increased to 0.0283 min^− 1^ with PANI/CeO_2_/TiO_2_ and ethanol, the influence of ethanol concentration on the photoelectrocatalytic degradation of TBBPA was optimized. The results are presented in Fig. 5b. The degradation efficiency reached a maximum value when the concentration of ethanol was 10 mmol L^− 1^, while the efficiencies gradually reduced with the increase of ethanol concentration. It indicated that addition of ethanol removed parts of holes and decreased the recombination rate of photogenerated electron-hole pairs, significantly enhancing the photoelectrocatalytic activity of PANI/CeO_2_/TiO_2_ NTAs.

The kinetics of the above reactions under simulated solar irradiation were studied and the results were displayed in Fig. 5. It can be seen that good linear relationships were achieved with the first order kinetic equation, and the linear correlation coefficients of these degradation experiments were in the range of 0.9959~0.9982, which clearly proved that TBBPA degradation reactions followed the first-order kinetics. Moreover, the kinetic constants exactly exhibited the effect of the annexing agent amount. Hence, 10 mmol L^− 1^ ethanol was added in the solution to enhance TBBPA degradation.

### Stability of the Photocatalyst

**Fig. 6:**
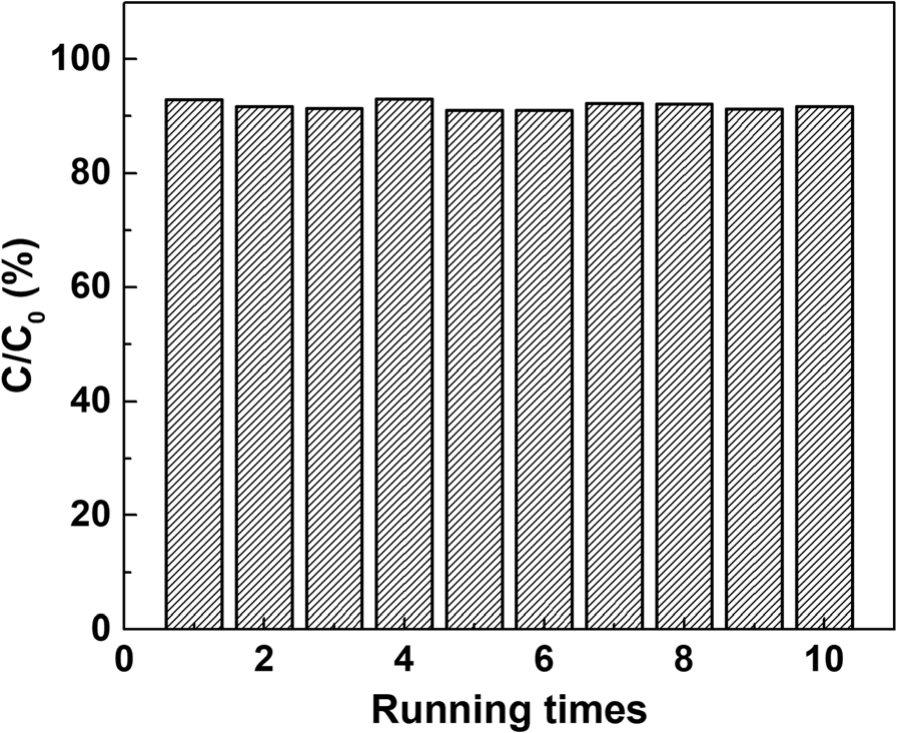
Reusability test of EC-PANI/TiO_2_ NTAs under optimal conditions

Figure 6 shows the degradation efficiencies of ten repeated runs of TBBPA degradation using PANI/CeO_2_/TiO_2_ NTAs with ethanol under the optimal conditions. The results showed that the degradation efficiencies of ten experiments were very close (< 3%) which indicated the prepared material had a good stability. As a consequence, PANI/CeO_2_/TiO_2_ NTAs could be reused for many times in the photoelectrocatalytic degradation towards TBBPA and be efficient catalysts with a high level degradation rate of 92%.

### Mechanism of Photoelectrocatalytic Degradation of TBBPA

Learned from the above experiments, CeO_2_ nanoparticles deposited on the surface of PANI/TiO_2_ NTAs were proved to significantly enhance the degradation efficiency of TBBPA. It was known that the photoelectrocatalytic oxidation of organic pollutants mainly attributed to the generation, transfer, and consumption of photogenerated electrons and holes in the interior of the TiO_2_ catalytic materials [32, 33]. In the photoelectrocatalytic degradation experiments towards TBBPA, PANI/CeO_2_/TiO_2_ NTAs were irradiated by simulated sunlight and produced photogenerated electrons and holes, which attributed to the activation of TiO_2_ and CeO_2_ by accepting photon energy (Eq. (1) and (2)). A part of generated electrons were transferred from both conduction bands (CB) of TiO_2_ and CeO_2_ to PANI. PANI coated on the TiO_2_ NTAs played a significant role to extend the absorption wavelength range, separate the charge rapidly, and inhibit the recombination of electrons and holes [34]. Another part of electrons was absorbed into CeO_2_ particles, and then Ce^4+^ ions of CeO_2_ were reduced to Ce^3+^, which could react with O_2_ and generate superoxide radical ion (•*O*_2_^−^) (Eq. (3) and (5)). At the same time, electrons could directly reduce O_2_ to form •*O*_2_^−^, which was a very reactive ion-radical and produced lots of hydroxyl radicals (HO•) and hydroperoxy radicals (HO_2_•) (Eq. (4), (8), and (9)). On the other hand, the photogenerated holes (*h*^+^) can react with H_2_O and OH^−^ to generate HO• and H^+^, which could be used in reactions (8) and (9). Finally, HO_2_• and HO•, which were regarded as the main active species in the PEC degradation procedure, as well as *h*^+^ directly react with TBBPA or the mediate products and thus the degradation process was accomplished (Eq. (10)). Hence, the possible mechanism for the photoelectrocatalytic degradation of TBBPA by PANI/TiO_2_ electrode could be expressed as follows:

1 $$ {\mathrm{TiO}}_2+ hv\to {\mathrm{TiO}}_2+\left({e}^{-}+{h}^{+}\right) $$

2 $$ {\mathrm{CeO}}_2+ hv\to {\mathrm{CeO}}_2+\left({e}^{-}+{h}^{+}\right) $$

3 $$ {\mathrm{Ce}}^{4+}+{e}^{-}\to {\mathrm{Ce}}^{3+} $$

4 $$ {\mathrm{O}}_2+{e}^{-}\to \bullet {{\mathrm{O}}_2}^{-} $$

5 $$ {\mathrm{Ce}}^{3+}+{\mathrm{O}}_2\to \bullet {{\mathrm{O}}_2}^{-}+{\mathrm{Ce}}^{4+} $$

6 $$ {h}^{+}+{\mathrm{H}}_2\mathrm{O}\to \mathrm{HO}\bullet +{\mathrm{H}}^{+} $$

7 $$ {h}^{+}+{\mathrm{OH}}^{-}\to \mathrm{HO}\bullet $$

8 $$ {\mathrm{H}}^{+}+\bullet {{\mathrm{O}}_2}^{-}\to {\mathrm{H}\mathrm{O}}_2\bullet $$

9 $$ 4{\mathrm{H}}^{+}+\bullet {{\mathrm{O}}_2}^{-}\to 2\mathrm{HO}\bullet $$

10 $$ {\mathrm{HO}}_2\bullet \mathrm{or}\ \mathrm{HO}\bullet \mathrm{or}\ {h}^{+}+\mathrm{TBBPA}\to \mathrm{degradation}\ \mathrm{products} $$

**Fig. 7:**
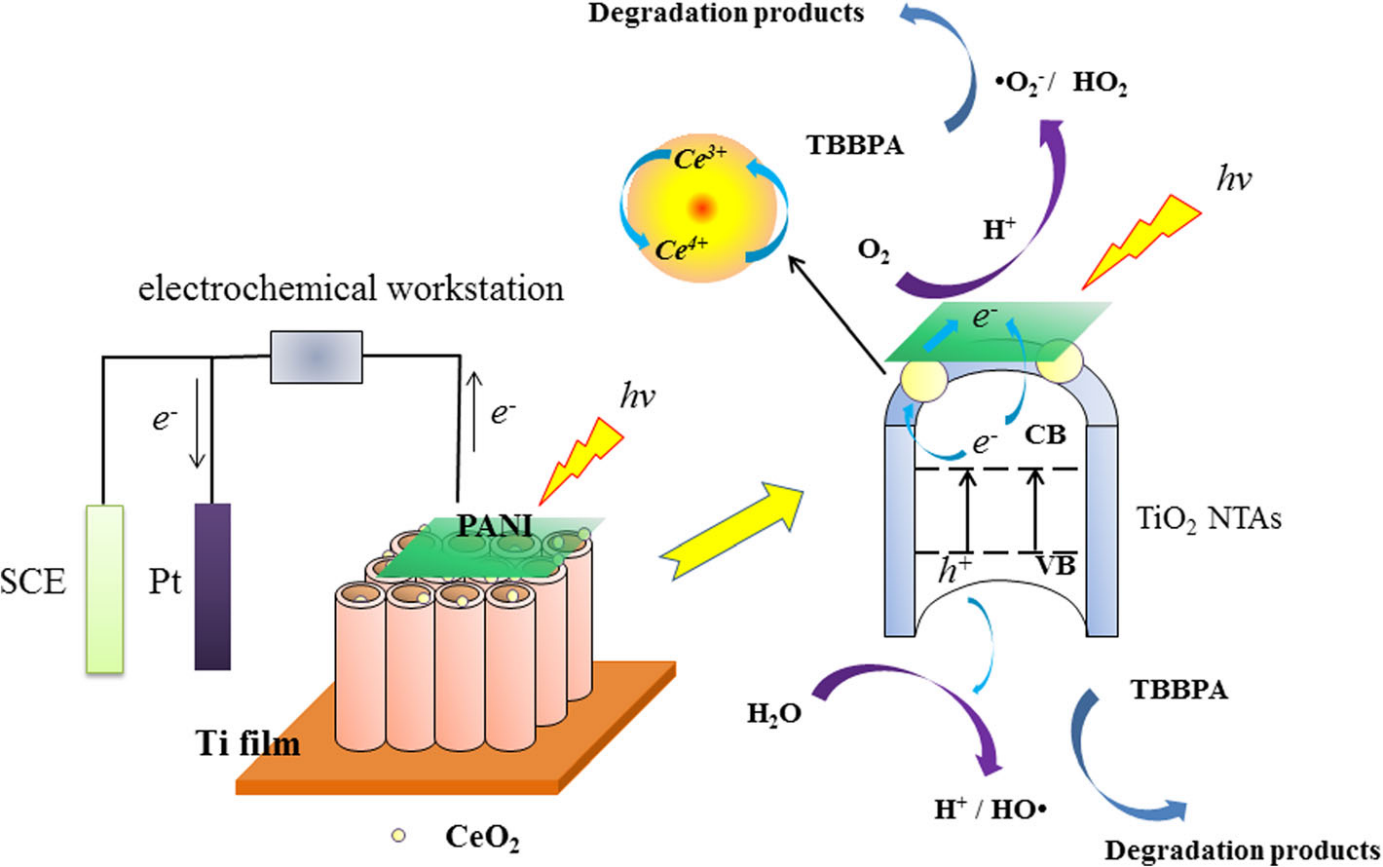
Degradation mechanism of TBBPA on PANI/CeO_2_/TiO_2_ NTAs under simulated sunlight

In a word, the PANI/CeO_2_/TiO_2_ NTAs are a good photoelectrocatalyst, and the possible degradation impacting factors were optimized and degradation mechanism was elucidated as shown in Fig. 7.

## Conclusions

PANI/CeO_2_/TiO_2_ NTAs were simply synthesized by an electrochemical method. PANI/CeO_2_/TiO_2_ NTAs exhibited extraordinary photoelectrocatalytic activity for the degradation of TBBPA with the assistance of ethanol. Under the optimum conditions, the degradation rate of TBBPA was higher than 92% in 120 min. The synergetic effect of PANI, CeO_2_, and TiO_2_ played a crucial role to increase the active free radicals, reduce the recombination rate of photogenerated electron-hole pairs, and enhance the catalytic performance. The degradation reaction followed the first-order kinetics. PANI/CeO_2_/TiO_2_ NTAs earned good reusability and stability. These results indicated that PANI/CeO_2_/TiO_2_ NTAs would be a promising catalyst for effective removal of TBBPA and some other organic pollutants.

## Abbreviations

BFRs: Brominated flame retardants
CB: Conduction band
EDS: Energy-dispersive X-ray spectroscopy
HO_2_•: Hydroperoxy radical
HPLC: High-performance liquid chromatography
PANI/CeO_2_/TiO_2_ NTAs: Polyaniline and CeO_2_ co-decorated TiO_2_ nanotube arrays
PEC: Photoelectrocatalytic
SEM: Scanning electron microscopy
TBBPA: Tetrabromobisphenol A
